# Eating out of Home: Influence on Nutrition, Health, and Policies: A Scoping Review

**DOI:** 10.3390/nu14061265

**Published:** 2022-03-16

**Authors:** Eva Gesteiro, Alberto García-Carro, Raquel Aparicio-Ugarriza, Marcela González-Gross

**Affiliations:** 1 ImFINE Research Group, Department of Health and Human Performance, Universidad Politécnica de Madrid, E-28040 Madrid, Spain; ag.carro@gmail.com (A.G.-C.); apariciougarriza.raquel@gmail.com (R.A.-U.); marcela.gonzalez.gross@upm.es (M.G.-G.); 2 Institute of Health Carlos III, CIBEROBN (Physiopathology of Obesity and Nutrition, CB12/03/30038), E-28029 Madrid, Spain

**Keywords:** diet, habits, eat out of home, health

## Abstract

Eating out of home (EOH) is a common practice worldwide but research gaps have been identified. The aims of this review were (a) to find a common definition for EOH, (b) to determine the nutritional contribution of EOH, and (c) to analyze the relationship of EOH with health parameters in adults. Fifty-seven articles were finally selected. The definition of EOH was not harmonized between researchers and the comparison between studies was quite difficult. Restaurant and fast food were the terms most used, followed by chain restaurant, à la carte, sit-down restaurant, eating at table, full service, ready to eat, takeaway, buffet and buffet by weight, bar, cafes, and cafeterias, either alone or attached to at least one of the above. The profile of the main EOH participant was a highly educated, high-income, and unmarried young man. EOH was related to a body mass index (BMI) or being overweight in a different way depending on age, sex, or EOH frequency. A high rate of EOH led to poorer diet quality, characterized by higher intakes of energy, total and saturated fats, sugar, and sodium, as well as lower intakes of fiber, dairy, fruit, vegetables, and micronutrients. Regarding beverages, a higher intake of soft drinks, sugar-sweetened beverages, fruit juices, beer, and other alcohol was observed when EOH. There is a need for a methodological consensus for analyzing the impact of EOH on dietary intake and health to avoid bias. Additionally, measures and policies should be utilized to help consumers to make healthier choices when EOH is compatible with business regarding those running EOH establishments.

## 1. Introduction

Eating out of home (EOH) is a widespread practice that was already done in ancient Rome when customers attended the tabernae and popinae [1]. Taverns, where mainly alcoholic beverages were served, started in the Middle Ages (XI–XII centuries). The oldest restaurant that is still open started in 1725 in Madrid, Spain (Casa Botín) [2] and the second oldest in Paris, France, was opened by the famous cook Boulanger in 1765. For Jean Anthelme Brillat-Savarin, author of “The Physiology of Taste,” which is the first gastronomic treatise on cuisine written from a philosophical point of view in 1825, the four requirements of a good restaurant were a distinguished atmosphere, friendly service, privileged cuisine, and an outstanding winery. These were characteristics that Boulanger lacked but possessed La Grande Taverne of London [3], run by Antoine Beauvilliers, former head chef of the Conde de Provenza. EOH was also related to the necessity to travel, according to the First Michelin Guide [2].

EOH has evolved during the centuries and the concept of fast food (FF) was introduced in the 1940 s of the XX century by the McDonald brothers. Nowadays, there are several concepts, such as bars, restaurants, lunch counters, snack bars, and buffets. EOH is becoming more frequent due to lifestyle changes [4,5], which is influenced by a complex range of social, individual, environmental, socioeconomic, biological, and/or psychological factors, as well as cultural singularities from each country or region [6]. In fact, EOH has a different meaning depending on the country. In some countries, EOH is the cheapest way of eating; therefore, it will be practiced by those people belonging to lower socioeconomic levels, while in other countries, it is a luxury. Inconsistency has also been observed regarding terminology and the definition of EOH. Most authors refer to restaurants when they try to define EOH, while others prefer to use the term restaurant next to FF to provide a definition of EOH [4,7,8,9].

Research has not grown in parallel to this tendency among the population in this area. The systematic review done by Lachat et al., in 2012 regarding EOH and dietary intake included only 29 studies, 11 performed only on adults. The topic has also been irregularly addressed depending on the country. Orfano et al. [10] stated in 2007 that more studies were available for the United States and a scarcity of studies was observed in Europe. In the already mentioned systematic review by Lachat et al., 11 studies were from the USA; 4 from the UK; 3 from Ireland; 2 from Kenya; 2 from Vietnam; and 1 each from Australia, Benin, China, and Belgium. Another three were related to the European Prospective Investigation into Cancer and Nutrition (EPIC) study. In fact, several articles were published as a secondary analysis of the EPIC data regarding EOH [10,11]. According to the Information Resources, Inc. (IRI) report 2017, in Europe, one in five meals (18% of all meals) are consumed outside the home [12]. More flexible menus and an increase of meal occasions from breakfast and all-day brunch through to dinner were observed. Likewise, the effect of health policies on people’s habits when EOH has been poorly studied [13].

As we identified several research gaps related to the topic of EOH, we aimed to perform a scoping review to critically address the following objectives: (a) to find a common definition for EOH, (b) to determine the nutritional contribution of EOH, and (c) to analyze the relationship of EOH with health parameters in adults.

## 2. Materials and Methods

### Search Strategy and Selection Criteria

To carry out this study, we examined the published literature studying the relationship between EOH with body weight and the prevalence of overweight and obesity, anthropometric data, socioeconomic factors, and diet quality (DQ). Medical Literature Analysis and Retrieval System Online (MEDLINE) and Web of Science [14] were used to carry out this scoping review. A search strategy was developed using keywords indexed by databases of specific terms related to EOH and following the approaches of a systematic review using Preferred Reporting Items for Systematic Reviews and Meta (PRISMA) guidelines. Publication dates were limited from 2009 to 2020 and the population considered were adults ≥18 years of age. The following terms were used for the search: Frequency of eating away-from-home OR take-away OR eat-out. For the Web of Science search, the following strategy was used: S = (((eat out of home) OR (eat away of home) OR (take away) OR (eat out) OR (frequency of eating away from home)) AND ((health) OR (BMI) OR (obesity) OR (overweight)) AND (adults)). The inclusion and exclusion criteria are presented in Table 1.

**Figure 1:**
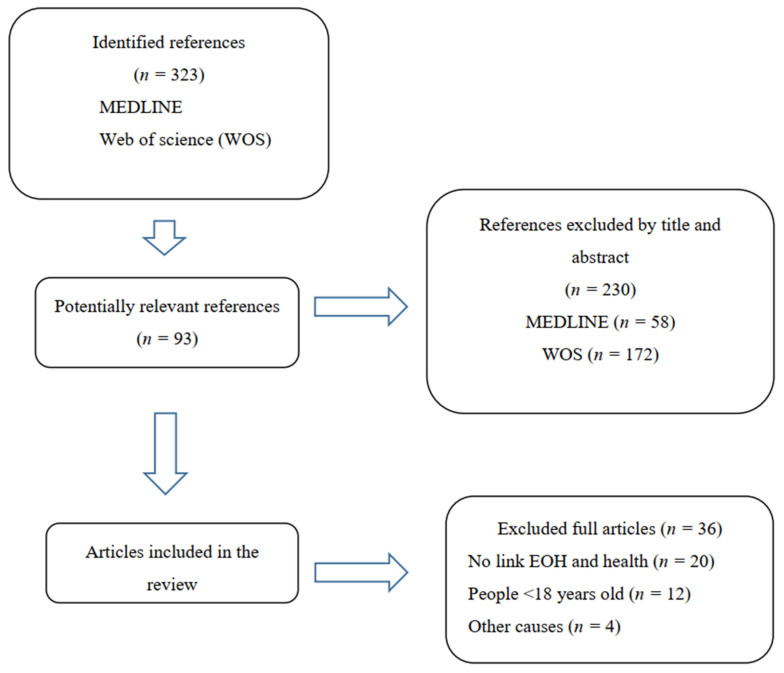
Flow diagram illustrating the screening process for eligible studies.

The literature search reported 323 articles. After an initial inspection by two independent reviewers (A.G.C. and E.G.), 230 publications were excluded based on the titles and abstracts. The 93 potentially relevant references were obtained as full texts. Of these, 36 references were excluded because they did not meet the inclusion criteria. The main reasons for the exclusion were that they did not seek a definition of EOH, did not provide nutritional information on EOH, or did not analyze the relationship between EOH and some health parameters, or the sample did not include adults ≥ 18 years old. The final references included were 57 articles. Figure 1 shows the flow diagram of the screening process.

## 3. Results

Studies included subjects aged between 18 to 93 years old. Samples sizes differed between (25) and (56,178). In total, data from more than 400,000 participants (56% females) were analyzed in this scoping review. Some authors did not include the total number of participants that they studied in their systematic or literature review.

Table 2 shows different types of restaurants and the countries where the studies were performed. After analyzing the 57 studies, we found no consensus regarding the definition of EOH. This analysis yielded several terms around which different investigations were conducted, all of which investigated the effects of EOH on health.

Table 3 displays the main findings linking EOH and nutritional outcomes. The main topics that were covered in this scoping review were: energy, macronutrient and micronutrient intake, types of beverages and foods, and diet quality. According to the number of retrieved articles, most attention was paid to dietary quality, energy intake, fat, fiber, and sodium intake. Regarding foods and beverages, most attention was paid to fruit, meat, dairy, and soft drinks.

## 4. Discussion

### 4.1. Definition for EOH

The term *restaurant* was the most used when defining EOH. Some studies considered only this place when EOH, while others used it in terms of FF, others *à la carte* or *sit-down* or *eating at a table* [31], and *full-service restaurants*. Among the 57 studies, 4 considered the term *restaurant* exclusively in its definition [4,7,8,9], while 14 articles had the term *restaurant* attached to others (FF, takeaway (TA), cafeteria, etc.) in the definition of EOH [15,19,20,27,31,34,35,36,37,38,39,40,41,42]. The high heterogeneity was confirmed in a previous systematic review published recently by Wellard-Cole et al. [56].

FF was another expression widely used in several definitions, either alone or sharing a definition with others. FF was defined by Grunseit et al. [22] and Larson [25] as cheeseburgers, hamburgers, pizzas, fries, and other foods that may not be commonly purchased out of home and EOH options such as meat pies, sausages rolls, roast, fried chicken, fried fish, and other TA options, such as Chinese, Thai, and Indian. Bes-Rastrollo et al., defined FF consumption as the sum of sausages, hamburgers, and pizza [52]. On the other hand, van der Horst et al., contemplated FF as a food category rather than a type of restaurant, as they noted that FF can not only be consumed as TA food but also within the restaurant. They defined FF as the consumption of products (TA and eating at a table) [31]. Duffey et al., demonstrated the concept using company names by attributing it to McDonald’s or Wendy’s [20]. Of the entire review, only three articles considered FF alone in their definitions of EOH [5,18,32], while 16 included the term in its definition that was also attached to other types of food services (cafeterias, restaurants, full service, etc.) [15,16,17,19,20,21,22,23,24,25,26,27,28,29,31,33].

Moreover, restaurant and FF were the terms most commonly used together by authors to define EOH, appearing in most of the articles of this review [4,5,7,8,9,15,16,17,18,19,20,21,22,23,24,26,27,29,31,32,33,34,35,36,37,38,39,40,42,57]. Other terminologies used to define EOH were chain restaurant, à la carte, sit-down restaurant, eating at a table, full service, ready to eat, TA, buffet and buffet by weight, bar, cafes, and cafeterias, either alone attached to at least one of the above.

Chain restaurant was not clarified by any author of the studies analyzed, though it appeared in the definition of Bleich and Pollak [17] and Robinson et al. [30]. The latter included all chains with 50 or more outlets in the UK; to categorize the chains as FF or full service, they used this definition of FF restaurants: “Restaurants that primarily provide consumers with largely pre-prepared ‘quick’ meals with little or no table service, with in-store seating and in which takeaway orders are likely to account for a significant proportion of orders.”

À la carte [43], sit-down restaurant [19,47], and eating at a table [31] were mentioned but none of them were well-defined. On the other hand, the term full service appeared in several articles next to restaurant [24,25,28,46] or next to FF without defining what it consisted of [29]. Two other terms found frequently were ready to eat and TA restaurants [23,26,45]. Gofee et al., studied eating TA meals at home [35], while McGuffin et al., studied the difference between the UK and the Republic of Ireland, where the latter considered TA together with restaurants [38]. Van der Horst et al., defined TA food as all foods consumed as TA, excluding FF [31]. Buffet [26,47] and buffet by weight [43] were two types of restaurants also considered by some authors. Cafeterias and bars belonged to the same group in several articles [15,16,22,27], while in eight of them, these terms were added to the definition, along with others, such as restaurant and FF [26,33,35,36,39,40,42,43].

Nago et al., included all definitions of EOH, while other authors focused on where food was prepared and consumed, distinguishing between where it was prepared/obtained [58] and consumed/eaten [45]. Therefore, the most coincidental terms in all definitions were restaurant and FF together and cafeteria, cafes, and bar together. Table 2 shows different types of restaurants and the countries where the studies were performed.

### 4.2. Nutritional Contribution of EOH and Their Relationship with Health Parameters in Adults

Some research concurred with each other in signaling that EOH was related to a poorer DQ [15,25,41,52]. In this sense, recent studies found that frequent restaurant meals were negatively related to DQ [56,59]. The associations between EOH, DQ, and overweight or obesity revealed that EOH was more prevalent among Hispanic/Latino adults, and it was associated with poorer DQ in the USA [26]. The same study obtained that EOH ≥ 1 time/week compared with EOH < 1 time/week was associated with lower odds of being in higher Alternate Healthy Eating Index-2010 (AHEI-2010) tertiles. As they argued, increasing EOH frequency was not associated with the odds of overweight or obesity, while eating from on-street vendors ≥1 time/week was associated with obesity.

Villacis et al., evaluated 19,371 Spanish participants who completed a food intake frequency questionnaire and showed an average carbohydrate quality index and fat quality index of 11.3 (on a scale from 4 to 20) and 1.7 (on a scale from 0.62 to 5.92), respectively. In their study, they ruled that the increase in the frequency of EOH ≥ 2 times a week was related to a poorer carbohydrate quality index and fat quality index [32]. Ziauddeen et al., claimed that leisure places, food outlets, and “on the go” combined contributed less energy from foods included in the principal food groups that are considered acceptable within a healthy diet (18%) than from other foods (30%), showing a dietary pattern that is incompatible with current guidelines in the UK [60]. Along this line, Larson detected that frequenting FF restaurants (burgers and fries) decreased healthy foods and key nutrients intake in young adults in the USA [25]. The authors of a recent study performed on American adults suggested that it was unlikely to meet all nutrient recommendations with any combination of dine-out menus, and thus fruits and dairy products should be consumed to maintain a balanced diet [51].

In the UK, food consumption patterns by eating location were different according to age groups. Most eating occasions (67–90%) involved eating food at home, with the percentage increasing with age. Work was the second-highest location category for eating occasions (except for those aged ≥65 years). The percentage of eating occasions at locations different from home and work were much lower in comparison and decreased with age for all locations, except leisure places, which remained comparable across all age groups [60]. American adults reported a mean of 3.9 EOH and 1.8 FF meals/week. More than 50% of adults reported ≥3 EOH meals/week and more than 35% reported ≥2 FF meals/week [37]. In China, the prevalence of EOH and at restaurants was 31.8% and 55.1%, respectively [41], where urban residents were EOH more than rural residents [42] and people living in neighborhoods with large numbers of indoor restaurants were more likely to engage in EOH [39].

Regarding breakfast, the effect of location was very strong. When EOH at restaurants/cafes for breakfast increased, the probability of including meat was higher than when EOH at work/college in the UK [36]. Grimes concluded that the individuals who ate breakfast at home had the highest DQ, while those who engaged in EOH for breakfast or skipped breakfast had DQ scores that were 3.98 and 4.62 points lower, respectively [21]. In China, urban residents were EOH at breakfast more than rural residents [42]. Another research observed that 25.0% of people who engaged in EOH did it at lunchtime, while 10.0% did it at dinner time [43].

Dinner eating locations were significantly associated with the nutritional DQ, both for the specific meal and whole-day intake. The data generally pointed to healthier dinners being consumed at home [50]. Lassen et al., showed that the overall DQ expressed as the energy density of food (excluding beverages) was significantly lower on days canteen takeaways were consumed compared to days when canteen takeaways were not consumed for dinner (average difference: −187 kJ/100 g) and daily (average difference: −77 kJ/100 g). Along the same line, significant differences were observed for intakes of fruits and vegetables (average difference: 83 g/dinner and 109 g/day, respectively) [57]. Orfanos et al., compared EOH versus food at home nutrient patterns among 27 centers in 10 countries and concluded that the composition of diet at home was different from that when EOH in southern European countries but was relatively similar in northern Europe. In northern Europe, EOH and food at home were homogeneous, whereas southern Europeans considered EOH as a distinctive occasion [27].

Foods in restaurants tended to be less healthy and more energy dense than those cooked at home [48,52]. Different studies concluded that higher habitual consumption of EOH and TA meals was associated with higher mean daily energy intake [25,35,45,61]. Robinson et al., analyzed main meals from 27 UK restaurant chains (21 full service, 6 FF) and they found that the energy content of all eligible restaurant meals (13,396 in total) was excessive, with 977 kcal per meal on average [30]. Full-service restaurants offered significantly more excessively caloric main meals, fewer main meals meeting public health recommendations, and on average, 268 (103 to 433) kcal more in main meals compared to FF restaurants. Eating at least one meal in restaurants was associated with an increase in daily total energy intake of 140 kcal compared with not eating at restaurants in Shanghai [41]. Conversely, Naska et al., found that EOH was weakly associated with total energy intake in eleven European countries [11]. Scientific literature showed an association between EOH and higher intake of total fat [5,15,25,41,45], saturated fat [15], trans fat [52], and cholesterol [51], and lower intakes of monounsaturated fats [52] compared to non-EOH. Orfanos et al., reported that EOH contributed more to total fat intake than to protein and carbohydrates intakes in European women [27].

Lee et al., analyzed the differences between non-home-made meals and home-made meals [61] in South Korea and noted that the non-home-made meals group obtained a higher percentage of energy (%E) from protein (23 vs. 15%) [45]. Regarding carbohydrates, EOH contributes more to sugar [5,27,48] and starch intakes [27] and is associated with a lower intake of dietary fiber [15,27,45,50,53]. Non-substantial out-of-home eaters (<25%) reported consuming proportionally higher quantities of sweet and savory bakery products when EOH than substantial out-of-home eaters (>25%E) in Europe [11].

EOH was associated with low levels of micronutrient intakes [51], phosphorus, potassium, niacin [45], and vitamin C [27,45]. Calcium was negatively related to breakfast EOH frequency in the Taiwanese population [54], while in southern Europe, EOH contributed less to calcium intake [27]. Recent studies showed that EOH was associated with a higher intake of sodium [24,42,53]. Furthermore, a current study found that those meals prepared out of home were related to inadequate intakes of dietary fiber, vitamin C, and several minerals among Japanese adults [62].

Focusing on the different food groups, we can conclude that fruits [5,16,24,52,53,55] and vegetables [5,52,53,55], milk [16] and dairy products [24,33,52], cereals [53], legumes [52], and olive oil [53] were less or rarely consumed when EOH in contrast with at home, while the intake of nuts [53], meat [16,36,52], and processed meat [52,53] increased. In Europe, substantial out-of-home eaters consumed higher amounts of essential food groups (meat, fish/seafood, vegetables, and potatoes) than non-substantial out-of-home eaters. The latter consumed higher amounts of indulging foods and lower amounts of essential food groups when EOH than at home [11]. Lee et al., differentiated between non-home-made and home-made and they found that non-home-made meals tended to include fried and grilled foods, had more one-dish meals, such as bibimbap, noodles, or dumplings, and showed a higher dietary diversity [45].

In a study conducted in the UK, EOH was found to increase the probability of consuming meat, as well as the amount consumed, compared to other situations (e.g., home, work) [36]. The authors analyzed the amount of meat eaten when included in a meal and noted that in the UK, this is likely to be greater in a restaurant/café, though EOH tends to reduce the amount of meat included, apart from the meat consumed at breakfast. A pattern of higher meat consumption in restaurants/cafes until 16:00 was observed, followed by lower consumption from 17:00 to 19:00 [36]. In some European countries, men reported higher intakes of fish when EOH than at home [11]. A study by Martins Rodriguez showed that the risk of being overweight/obese was 11% higher in consumers who did not choose rice and beans than in those who did [47]. In fact, a scientific report suggested a weekly consumption of 1.5 cups of legumes (beans and peas) [63].

Regarding beverages, Myhre [50] and Bes-Rastrollo et al. [52] concluded that restaurant dinner customers used to drink alcohol in a high percentage. According to a study performed in Italy, those who reported EOH at any time had higher intakes of beer [53]. Larson detected that frequenting FF restaurants (burgers and fries) increased sugar-sweetened beverages intake [25]. Non-substantial out-of-home eaters reported consuming proportionally higher quantities of soft drinks [11,16], juices, and other non-alcoholic beverages when EOH than at home [11].

Sex, income, frequency of EOH, and frequency of drinking are significant factors that affect consumption patterns [60]. Moreover, it is important to mention that this scoping review showed a wide analyzed sample range. Dave et al., found that the frequency of FF intake decreased with age [18]. Age was also found to be significantly negatively related to FF being perceived as unhealthy and a dislike toward cooking. Some studies concluded that highly educated, high-income, and unmarried young men were linked to EOH [11,18,33,43,64]. Nevertheless, it was weakly associated with total energy intake [11], while Wellard-Cole et al., found a high energy and nutrients intake in young males [56]. Bes-Rastrollo et al., reported that EOH consumers were younger, likely to be smokers, and presented higher baseline weight and body mass index (BMI) [52]. Mills found that a higher frequency of home-made food eating was associated with being female, older, of higher socioeconomic status, and not working overtime [64]. Choi et al., noted that choosing healthy meals when EOH is more important for housewives than refraining from EOH [4]. FF and TA were associated with gender and age, which were also influenced by different lifestyle determinants [31]. Compared to the individual behavior variables, the identified lifestyle patterns found by Fuglestad et al., appeared to be more reliably related to diet, physical activity, and body weight [5]. As for the perception of how EOH affects health, Dave et al., observed that FF was perceived to be less unhealthy with an increasing number of people and children living in the household [18]. In a study conducted by Grunseit et al., participants cognitively recast TA food consumption as negative (expensive and unhealthy) and considered reducing consumption of such foods or consuming healthy alternatives as a (positive) self-care action [22]. It was very likely that motivations related to time, effort, and cooking were of increasing importance for food choices in our society [31] and, though some consumers were aware of some health consequences, healthy eating was not a priority when EOH because it was related to special occasions [7].

Environmental and socioeconomic factors should be considered to have a clearer idea of the mechanisms that lead to EOH [15,48]. Choi et al., also agreed that the relationship between the frequency of EOH and the total amount of energy intake depends on the socioeconomic status [4]. FF was preferred among women with low socioeconomic characteristics, whereas non-FF restaurant meals were more likely to be frequently consumed among women with high socioeconomic characteristics [65]. Kasparian et al., compared parenting practices and decision making at restaurants vs. at home [9]. Authors noted that mothers reported more permissive food rules at restaurants, and they were more likely to make decisions about whether they eat out, where to eat, and children’s meal selections than their children. Furthermore, TA was frequently related to lower socioeconomic status, whereas EOH was more frequently associated with higher socioeconomic status and working overtime. Sociodemographic characteristics associated with the frequency of EOH varied with the meal source [64]. Lee et al., claimed that eating home-made food did not necessarily guarantee a healthy diet, and the effects of meal preparation location on nutritional status might vary depending on sociodemographic characteristics [45].

Data regarding physical activity are controversial, where some authors reported that people who engage in EOH more frequently practiced less PA [5], while others stated that EOH consumers were more physically active [52]. Non-home-made meals were linked to an increase in abdominal obesity and obesity among women, irrespective of the types of PA. Dietary intake may differentially influence waist circumference [66] for women compared with men. The separately significant associations between increased consumption of non-home-made meals (sedentarism, higher frequency of TV watching) with obesity prevalence increased women’s risk for obesity-related chronic diseases. However, the model used was adjusted for various types of PA, attenuating these associations. This study suggested PA benefits for women, even when it is moderate [51].

There is also observed the impact of EOH on the prevalence of metabolic syndrome, overweight, and obesity. In this regard, EOH increased the risk of metabolic syndrome for middle-aged males (45–60 years) and higher abdominal adiposity and blood pressure for older women, while for both younger men and women (<45 years), the risk of elevated blood pressure decreased [40]. Choi et al., observed that the waist-to-hip ratio, serum total cholesterol [67], and cholesterol transported by low-density lipoproteins were significantly higher among participants who rarely dined out of home than those who did not [4]. Some authors concluded that EOH and FF meals were adversely associated with metabolic health outcomes, such as blood triglycerides or cholesterol transported by high-density lipoproteins [20,37] In fact, it was suggested that reducing FF consumption could be an important goal for preventing adverse outcomes for metabolic health [20]. Nevertheless, a recent study in older adults showed that those who ate prepared meals had a better DQ compared with when they did not receive the food from the Older Americans Act Nutrition Program [68].

Some authors have not found any association between BMI [18,49], obesity [4], and EOH or FF. Maybe these findings could be explained by the differences in the type of restaurants considered [52]. Conversely, other authors suggested that this relationship exists. Regarding EOH, some studies concluded that people having frequent EOH had elevated BMI [8,24,37,39,42,55] and it was positively associated with the risk of being overweight or obese and changing weight [52,58]. Weight status in the previous 5 years influenced EOH and weight gain. Thus, those who gained ≥3 kg in the previous 5 years showed a statistically significant association between EOH and weight gain, while those with weight losses of ≥3 kg displayed a lower magnitude for the association without significance [52].

EOH frequency at restaurants and TA predicted positive BMI changes in women [58]. In a study performed by McClain [26], almost half of American participants (47.1%) reported EOH ≥ 5 times/week, where more than one-third (37.2%) were classified as overweight and 39.6% as obese. In this sense, the last report from the United States Department of Agriculture in 2018 showed that FF contained higher levels of saturated fats and sodium and less calcium, iron, and fiber than food cooked at home [13]. Likewise, FF contains fewer vegetables and fruits and overall has more calories. For these reasons, recent legislation aims to help consumers to choose healthier FF and present more healthy options [13].

Some studies performed in China showed that EOH influenced BMI and waist circumference among males more than among females [8,39]. The effects of EOH on BMI were observed in urban areas but not in rural ones, which could be explained by the different energy expenditures due to the different labor intensities [42]. Around 27% of the studied population reported an EOH frequency of ≥2 times/week. EOH consumers had a statistically significant higher average weight gain and BMI gain, and a higher risk of gaining ≥2 kg/year during the 4-year follow-up, without differences in the prediction of weight gain between sexes [52]. Contrariwise, EOH contributed to an increase in overweight and obesity, mostly among men, as women made healthier choices when sitting, and it may be a protective factor against obesity for them [49]. Some studies showed that people having frequent FF meals had elevated BMIs [37] in the USA, body weight and waist circumference [58] in several countries, and increased risk of overweight and obesity [25] in the USA; meanwhile, no significant association was found between heavy EOH at FF restaurants and BMI or waist circumference in South Korea [24]. De Castro et al., obtained that compared to food at home, overweight and obese participants had larger meals when EOH than normal-weight participants [19]. A recent study observed that frequent consumption of meals cooked away from home was significantly related to an increased risk of all-cause, cardiovascular, and cancer mortalities [69].

### 4.3. Healthy Policy Implications

Public policies are needed to help EOH consumers make healthier choices that consider the EOH trends according to different regions of the same territory and educational and socioeconomic levels [48,49]. Public health interventions should focus on the availability and access to healthy foods, promotion of healthy food choices, and behaviors across multiple locations, environments, and contexts for food consumption [60]. Du et al., also argued that public health initiatives are needed to encourage Chinese adults to make healthy food choices when EOH [8]. Along this line, programs and policies could improve the healthfulness of FF outlets [20] and the nutritional quality of affordable EOH choices [24]. Grunseit et al., found that some participants actively created social environments supportive of healthy choices [22]. The identified strategies were consistent with documented techniques for successful behavior change and corresponded to all levels in the social model from intrapersonal factors to public policy. The findings could underpin health promotion strategies to support this at-risk group.

More interventions to promote healthier food choices among young adults who reported frequent burger-and-fries restaurants use are needed [25]. Martins Rodriguez showed that efforts with helping consumers make healthier food choices when EOH and thereby possibly decreased weight gain should address those aspects, along with sociodemographic factors [47]. The findings of Kasparian et al., suggested that parenting practices toward overall behavior and food choices may differ between restaurants and food at home, highlighting the importance of healthy menu options, further research, and educational strategies [9]. Some authors agree that a greater recognition of public health and nutritional education aimed at promoting healthy eating choices was necessary.

The interest in the publication of calories EOH differed significantly by sociodemographic characteristics [17]. More than three-quarters of the participants (76%) stated that the knowledge of food calories in a restaurant would be useful, 68% felt that the government should encourage restaurants to publish caloric information on menus at the point of purchase, and 51% were more likely to eat in a restaurant that reported calorie information on menus. Regarding consumer nutritional knowledge (NK), most participants knew daily energy requirements for moderately active men and women but tended to underestimate those for inactive adults [17].

Larson et al. [25] suggested that as legislation requiring chain restaurants to list calorie information on the menu was going to come into effect soon, it would be important to assess whether calorie labeling helps young adults to choose nutrient-dense menu options. Time elapsed since then has demonstrated this measure to be less successful than estimated. This fact is still controversial, as other authors found menu labeling effective in reducing energy ordered and consumed [70]. A study performed with calorie-unlabeled McDonald’s menus showed that it was not associated with changes in calories purchased in adults, adolescents, or children [71]. Although participants were more likely to notice calories on menus post-labeling, there was no improvement in the ability to accurately estimate calories purchased. Some authors suggested that improving NK in people at risk of chronic diseases can be a useful tool for prevention [54].

An important aspect is how restaurants can maintain their benefits by also offering healthy menu choices among their customers. Hillier-Brown et al., defined “signposting” as the addition of a symbol to menus identifying “unhealthy” main dishes and found a decrease in unhealthy main dishes ordered, while there was no significant overall change in sales of all “healthy” items [23]. A price reduction for healthier choices resulted in a proportional increase in sales of healthier items compared to other items.

The importance of availability and convenience as effective tools to promote healthy eating habits reinforces the idea that providing healthy TA dinners had the potential to promote healthy dietary habits among employees [57]. Along the same line, English et al., found that the food environment variables, such as convenience, meal characteristics, social influences, food type, and nutrient content, as well as the ED and palatability, also influence the strength of the size portion effect [34]. Changes in the number of eating occasions, energy-dense food, and portion size were shown to be main contributors to changes in total energy intake in the USA [60].

It was confirmed that variables such as sex, age, income per capita, educational level, total energy intake, smoking and/or drinking status, residential area, and PA were key in developing strategies to prevent metabolic syndrome in different populations [40,72]. Similarly, interventions seeking to reduce energy content through reformulation or diminishing portion sizes in restaurants, cafes, and TA could potentially lead to a decrease in mean daily energy intake [23,28,35]. As mentioned above, taking these measures, as well as “signposting,” compatible with an economic benefit is a challenge, which did not always generate a significant change in sales [23]. An increase in price for unhealthy choices when combined with “signposting” decreased unhealthy main dishes ordered [16,23].

Another condition regarding the prevalence of obesity was the existence or not of FF outlets. Fraser et al., summarized whether the availability of foods high in fat, salt, and sugar through FF or TA outlets was involved in the causal route to epidemic obesity and highlighted areas for future work [46]. A Canadian study observed that these kinds of meals had high levels of fat, saturated fat, sodium, and sugar [68]. The association between FF consumption and exposure to FF outlets found conflicting results that may be due to a lack of information about good diet quality.

The main limitation of this review was the lack of consensus in the definition for EOH; however, a wide discussion was performed with all the results found. There are many types of EOH depending on the countries, which increased the difficulty of the analysis of the health outcomes. Nevertheless, this scoping review aimed to analyze all the related health aspects in adults. Another limitation was the impossibility to perform a systematic review, as some studies were an analysis of the policies (i.e., without participants) or they were literature reviews. For that reason, this was a scoping review that aimed to identify key aspects of EOH on adults’ health. It is important to transmit to governments, stakeholders, and authorities that a healthy variety of EOH is necessary.

## 5. Conclusions

EOH has different meanings depending on the country, as types of restaurants also vary. Harmonizing the definition of EOH is not easy and, consequently, extrapolating research on EOH from one country to another is not always possible. The profile of the main out-of-home eater is a highly educated, high-income, unmarried young man.

A high frequency of EOH leads to a lower DQ, characterized by a high energy intake, an excess of total and saturated fats, sugar, and sodium, as well as low amounts of fiber, dairy, fruit, vegetables, and micronutrients. Regarding beverages, EOH was associated with a higher intake of sugar-sweetened beverages, soft drinks, fruit juices, beer, and alcohol. EOH appears to be related to BMI, overweight, and some components of metabolic syndrome. The effects of EOH depend on aspects such as age, sex, frequency of EOH, and the country studied. There is a need to promote health policies to help consumers to make healthier choices when EOH, mainly via healthy food availability, which should be compatible with business.

## Figures and Tables

**Table 1:** Data extraction and criteria for the evaluation of the included studies.

Inclusion Criteria	Exclusion Criteria
≥18 years old	≤18 years old
Definition of EOH and evaluation of EOH with health parameters	Schools and/or educational interventions, COVID-19 studies
Studies that investigated EOH and its association with health	Studies that did not investigate the association between EOH with health

EOH: eating out of home.

**Table 2:** Grouping of eating out of home by type of establishment and country.

Type of Establishment	Country (Number of Articles)	Total Number of Articles	References
Fast food	United States of America (12) Australia (1) United Kingdom (2) South Korea (1) Greece (1) Switzerland (1) Spain (1)	19	[5,15,16,17,18,19,20,21,22,23,24,25,26,27,28,29,30,31,32,33]
Restaurant	Australia (1) South Korea (1) China (4) United States of America (6) United Kingdom (3) Greece (1)	16	[4,7,8,9,15,19,20,27,34,35,36,37,38,39,40,41,42]
Cafeterias and bars	United States of America (2) Australia (1) Greece (1) Brazil (1) United Kingdom (2) South Korea (1) United States of America (1) China (3)	12	[15,16,22,26,27,33,35,36,39,42,43,44]
Ready to eat and take away	United Kingdom (3) United States of America (1) South Korea (1) Switzerland (1)	6	[23,26,31,35,38,45]
Full service	United Kingdom (2) South Korea (1) United States of America (2)	5	[24,25,28,29,46]
Buffet and buffet by weight	Brazil (2) United States of America (1)	3	[26,43,47]
Sit-down restaurant	United States of America (1) Brazil (1)	2	[19,47]
Chain restaurant	United States of America (1) United Kingdom (1)	2	[17,30]
À la carte	Brazil (1)	1	[43]
Eating at the table	Switzerland (1)	1	[31]

**Table 3:** Main findings linking eating out of home and nutritional contribution.

Topic Regarding EOH	Finding	References
**Energy**		
Energy intake Energy dense foods	High High	[25,35,41,45,48,49]
**Macronutrients intake**		
Protein	High	[45]
Fat	High	[5,15,25,41,45,50]
Saturated fat	High	[15]
Cholesterol	High	[51]
Trans fat	High	[52]
Monounsaturated fat	Low	[52]
Fiber	Low	[15,27,45,52,53]
**Micronutrients intake**		
Micronutrients	Low	[51]
Sodium	High	[41,51,53]
Phosphorus	Low	[45]
Potassium	Low	[45]
Niacin	Low	[45]
Calcium	Low	[27,54]
Vitamin C	Low	[27,45]
**Beverages intake**		
Dairy, milk	Low	[16,24,52]
Soft drinks	High	[11,16,52]
Fruit juice	High	[11,52]
Sugar-sweetened beverages	High	[25]
Beer	High	[53]
Alcohol	High	[50,52]
**Food intake**		
Sugar	High	[5,27,48]
Starch	High	[27]
Fruit	Low	[5,16,51,52,53,55]
Vegetables	Low	[5,52,55]
Cereal	Low	[53]
Legumes	Low	[52]
Olive oil	Low	[53]
Nuts	High	[53]
Meat	High	[16,36,52,53]
Fish	High	[11]
Bakery	High	[11]
**Food**		
Diet quality	Poor	[15,25,26,32,41]

EOH: eating out of home.

## Data Availability

No new data were created or analyzed in this study. Data sharing is not applicable to this article.
